# Parkinson’s Disease in Light of the COVID-19 Pandemic

**DOI:** 10.3390/brainsci12020143

**Published:** 2022-01-21

**Authors:** Anna Drelich-Zbroja, Mateusz Cheda, Maryla Kuczyńska, Izabela Dąbrowska, Ewa Kopyto, Izabela Halczuk

**Affiliations:** 1 Department of Interventional Radiology and Neuroradiology, Medical University of Lublin, 20-059 Lublin, Poland; mateusz.cheda@gmail.com (M.C.); maryla.kuczynska@gmail.com (M.K.); izis608@gmail.com (I.D.); 2 Students’ Scientific Society at the Department of Interventional Radiology and Neuroradiology, Medical University of Lublin, 20-059 Lublin, Poland; ewa.kopyto@gmail.com (E.K.); halczuk.izabela@gmail.com (I.H.)

**Keywords:** COVID-19, SARS-CoV-2, Parkinson’s disease, parkinsonism

## Abstract

In this review we attempt to collate the existing scientific evidence regarding the possible role of severe acute respiratory syndrome coronavirus 2 (SARS-CoV-2) in the pathophysiology of Parkinson’s disease (PD), as well as to investigate the impact of PD/parkinsonism on the clinical course of the viral infection itself. Since etiology of PD is not completely understood, various studies suggest different potential links between coronavirus disease 2019 (COVID-19) and PD. Suggested connections include, among others, similar prodromal symptoms, renin–angiotensin–aldosterone system involvement, or gut microbiome dysbiosis participation. Despite the initial assumptions that, as a mainly elderly population suffering from rigidity of respiratory muscles, impairment of cough reflex, and dyspnea, PD patients would be more susceptible to viral infection, and would experience a more aggressive course of COVID-19, the published scientific reports contain mutually exclusive data that require further investigation and meta-analysis.

## 1. Introduction

Since the epidemic began in December 2019, the newly reported coronavirus - severe acute respiratory syndrome coronavirus 2 (SARS-CoV-2) has been widely studied in terms of its effects on multiple organs, primarily respiratory and central nervous systems (CNS). The virus’s targeting of various cell types has been partially attributed to the fact that SARS-CoV-2 utilizes angiotensin-converting-enzyme 2 (ACE2) type 1 transmembrane receptors localized on the epithelial and endothelial cells of the respiratory system, as well as on immune cells, to enter and replicate within the human organism. Interestingly, ACE2 receptors are widespread within brain structures, the central nervous system, and visual tracts, which may explain the many neurological symptoms in coronavirus disease 2019 (COVID-19) infection [1,2,3]. Interaction with ACE2 receptors of the vascular endothelial cells leads to disruption of the blood–brain barrier, with consequent cerebral edema and microhemorrhages. In addition, SARS-CoV-2 may exert direct neuronal damage due to the affinity of the spike S1 protein of the virus towards ACE2 receptors expressed on neurons. There are two possible pathways of brain invasion, either blood-borne or through retrograde axonal transport (olfactory route) [2,3]. Brain tissue may be exposed to injury as a result of COVID-19-related hypercoagulable states, hypoxia, inflammation and immune response (cytokine storm), or dyselectrolytemia as well [2,3]. It is these events that are currently thought to play the major role in the cerebral pathology reported during infection, as mounting evidence shows negligibly low levels of both viral RNA and proteins in the brains of affected patients, making the direct pathomechanism of viral damage an unlikely cause of all the neurological symptoms [4,5]. Nonetheless, the interplay between SARS-CoV-2 and abundant ACE2 receptors gives solid grounds to discuss the role of COVID-19 in neurodegenerative conditions. Recent studies indicate that there may exist (not yet elucidated) a molecular link between the infection and the pathogenesis of Alzheimer’s disease, multiple sclerosis, and Parkinson’s disease (PD)/parkinsonism [1,3,6,7,8,9].

In this review we attempt to collate the existing scientific evidence regarding the possible role of SARS-CoV-2 in the pathophysiology of PD/parkinsonism, as well as to investigate the impact of PD on the clinical course of the viral infection itself.

## 2. The Neurotropic Potential of SARS-CoV-2 and Its Possible Role in the Pathogenesis of Parkinson’s Disease/Parkinsonism

Prior to the COVID-19 pandemic, multiple neurotropic viruses were already recognized, i.e., herpes simplex virus (HSV), poliovirus, influenza A virus, and representatives of the Coronaviridae viral family [9,10,11]. Viral RNA and proteins identified in the brain specimens during post-mortem examinations of patients diagnosed with COVID-19 strongly suggest neurotropic characteristics of SARS-CoV-2 as well [12,13]. The association of parkinsonism with viral infections has also been documented in the past. The most commonly cited example of such a relationship is post-encephalitis parkinsonism during the pandemic of Spanish Flu (influenza A virus H1N1) in 1918 [9,14]. Almost all patients who suffered from encephalitis in the course of influenza virus infection developed symptoms of parkinsonism. In addition, a 2–3-fold increase in the risk of developing parkinsonism in people born between 1888 and 1924 was noted [11,14,15]. Ayele et al. presented four cases of SARS-CoV-2 infection-associated parkinsonism in patients who had no previous history of prodromal symptoms of PD. All patients experienced hypo/anosmia, two developed symptoms of encephalitis, and bilateral pallidal lesions were found in the MRI of one patient [16]. Ghosh et al. described a case of parkinsonism following osmotic demyelination syndrome in the course of SARS-CoV-2 infection in an elderly diabetic woman [17]. Tien Lee Ong et al. published an interesting case report of the COVID-19 associated acute necrotizing encephalopathy manifesting as parkinsonism with myorhythmia [18].

The etiology of PD is not completely understood [3,19]. Currently, it is believed that PD affects numerous systems of the human organism [3,20]. Main symptoms of the disease develop predominantly due to the damage or death of nigrostriatal neurons in the brain and the depletion of striatal dopamine stores [3,9,12,20]. One of the leading hypotheses suggests that aggravation of oxidative stress, resulting from the accumulation of excessive reactive oxygen species, leads to a build-up of the alpha-synuclein protein in the form of cytoplasmic aggregates known as Lewy bodies [3,21,22]. A concurrent dual-hit hypothesis is postulated, where the first ‘hit’ is described as the penetration of an ‘unknown pathogen’ into the brain via the olfactory system or the gastrointestinal tract. The pathogen activates glial cells, making the brain more susceptible to oxidative stress, accelerating the brain aging process, and stimulating neurodegeneration. Some studies hypothesize that SARS-CoV-2 may be such a pathogen [11,23].

It is suggested that SARS-CoV-2 neuroinfection leads to elevated alpha-synuclein levels [6,24]. Alpha-synuclein is a presynaptic protein expressed among others in the substantia nigra and thalamus and is one of the major constituents of Lewy bodies. Studies have shown that alpha-synuclein protein aggregation (along with oxidative stress and glial cell activation) in the course of PD contributes to the loss of voluntary motor control due to degeneration of nigrostriatal dopaminergic neurons [25]. Moreover, the link between mutations of the alpha-synuclein gene (duplications and triplications) and familiar PD is well known [26,27,28]. Similar observations were previously made with regard to infection with the West Nile and Western Equine Encephalitis viruses. Evidence shows that animals with lower levels of neuronal alpha-synuclein were more likely to develop encephalitis induced by the West Nile virus. In turn, alpha-synuclein may have an inhibitory effect on virus spread [29,30]. Experimental studies showed that a persistently elevated level of alpha-synuclein promotes the formation of aggregates that are characteristic for neurodegenerative diseases including PD [29,31]. Semerdzhiev et al. identified a SARS-CoV-2 protein that promotes aggregation of the alpha-synuclein in a test tube [6]. The acceleration of the onset of alpha-synuclein aggregation was observed in the presence of the SARS-CoV-2 nucleocapsid protein (N-protein). This may suggest the possibility of future development of parkinsonism/PD symptoms in COVID-19 recovery patients [6].

The systemic inflammatory process and so-called cytokine storm tend to play a major role in both neurodegeneration and SARS-CoV-2 infection. Studies report that SARS-CoV-2 enters the cells interacting with ACE2 receptor and transmembrane serine protease-2 (TMPRSS2) [3,32,33]. As a result, serum angiotensin II levels are elevated, leading to the activation of inflammatory cytokines, including interferon-gamma, and aggravating the cytokine storm [3,34,35]. Increased levels of interleukin-1beta (IL-1beta), interferon gamma, interferon-induced protein 10, and monocytic chemoattractant protein 1 were observed in severe cases of COVID-19. IL-1, IL-2, IL-6, and tumor necrosis factor-alpha (TNF-alpha) are also involved in the cytokine storm, and their effects on the central nervous system were shown in the past [36,37].

Apparently, a direct link also exists between COVID-19, the renin–angiotensin–aldosterone system (RAAS), and PD. Several studies pointed out the role of ACE in PD [3,38]. Patients treated with ACE inhibitor–perindopril showed improved motor responses to dopaminergic precursor 3,4-dihydroxy-1-phenylalanine [3,38,39]. In animals, model ACE inhibitors were shown to protect from the loss of dopaminergic neurons [39,40].

Neuropathological studies using immunostaining of alpha-synuclein aggregates suggest that PD begins in either the olfactory or intestinal nerves and spreads to the brain. Common COVID-19 symptoms include hyposmia and hypogeusia (which are also prodromal symptoms of PD), indicating that SARS-CoV-2 has direct access to brain regions relevant for PD [10,12,14].

Another theory exists, which claims that the inflammatory process leading to PD is initiated by gut microbiome. The imbalance of gut microbiome or immune system deficits can lead to gut dysbiosis. Local inflammation promotes the increased gut permeability and bacterial translocation to the bloodstream and to remote organs such as the brain. Bacterial products trigger the disruption of the blood–brain barrier, resulting in neuroinflammation. This generates a loop in which peripheral immune cells are recruited and activated, further promoting neurodegeneration [41]. Growing evidence supports altered gut microbiome, described as dysbiosis in the bacterial microbiome and mycobiome, in patients with COVID-19 compared to the healthy control group [42,43,44].

Clira et al. in a community-based case–control study on the symptomatic effects of COVID-19 on PD showed a significant impact of SARS-CoV-2 on the deterioration of both motor (and motor disability) and non-motor PD symptoms [45]. However, authors speculate that non-motor symptoms of non-severe COVID-19 are caused by systemic inflammatory response rather than by neuroinfection. This suggestion requires further studies in a larger population.

Erro R. reported two cases of severe dyskinesia (one of the typical symptoms of parkinsonism) after receiving the BNT162b2 (Pfizer/BioNTech; Pfizer, New York, NY, USA; BioNTech, Mainz, Germany) mRNA vaccine. Both patients were > 60 years old females, previously diagnosed with PD (5 and 11 years prior to vaccination). Authors suggested that the observed symptoms could had been triggered by systemic inflammatory response [46].

Given all the above, one may have an impression that multiple unrelated theories exist regarding possible links between COVID-19 and neurodegeneration. However, to some extent all the concepts tend to complement each other. The N-protein of SARS-CoV-2 was shown to accelerate the aggregation of alpha-synuclein in vitro. Such a response in a living organism might be considered a natural protection against virus spread, as previously reported, based on the experience with the West Nile virus. At the same time, the accumulation of alpha-synuclein within the nigrostriatal dopaminergic system results in neurodegeneration, clinically visible as typical PD/parkinsonian symptoms. Therefore, an assumption can be made, that the SARS-CoV-2 invasion of the CNS may result in comparable motor symptoms. The second aspect is the possible route of entrance for SARS-CoV-2; apparently, the virus utilizes the widespread ACE2 transmembrane receptors, which may be encountered both within the olfactory route and intestinal nerves, common primary locations of alpha-synuclein aggregates in both PD and COVID-19. Relatively large data support the possible role of the cytokine storm and the inflammatory response in pathogenesis of the aforementioned conditions. Once more, it is the renin–angiotensin system that is involved in initiating the cascade of proinflammatory cytokines. To the contrary, a better response to dopamine analogues was observed in PD patients treated with ACE inhibitors; in animal models ACE inhibitors protected against the loss of dopaminergic neurons. Although all these assumptions are rather circumstantial in nature, a dual-hit hypothesis, in which SARS-CoV-2 may play the initiating role in the pathomechanism of PD symptoms, seems probable.

## 3. The Interplay between Parkinson’s Disease/Parkinsonism and COVID-19—Does One Entity Affect the Symptomatology of the Other?

PD is a neurodegenerative disorder of the central nervous system that mainly affects the motor system, the nigrostriatal pathway in particular. Therefore, it is of no surprise that major PD symptoms include tremor, rigidity, bradykinesia/akinesia, and postural instability. However, the clinical picture also encompasses non-motor symptoms (NMSs) such as anxiety, dementia, depression, and fatigue, among others [47].

The COVID-19 pandemic has had a negative, indirect impact on patients with PD/parkinsonism. This may be explained by the hypothesis of dopamine-dependent adaptation. The pandemic has changed daily life and routine, and flexibility in cognitive (and motor) functions is required to adapt to these changes. In patients with PD, both cognitive and motor flexibility is lower or even absent, which is the result of damage to nigrostriatal dopamine neurons. Such patients experience confusion and increased psychological stress, which can lead to the worsening of parkinsonism symptoms as well as mental illnesses such as anxiety and depression. The COVID-19 pandemic also caused a decrease in physical activity and made access to medical care more difficult, which may have contributed to an increase in motor and non-motor parkinsonism symptoms [48,49].

Studies have suggested that patients with PD or parkinsonism may have an increased risk of COVID-19, mainly due to the fact that PD most often affects elderly people with numerous comorbidities and multidrug therapy [50]. Moreover, respiratory muscle rigidity, impairment of cough reflex, and dyspnea are not uncommon in the course of parkinsonism, implying these symptoms can be the possible cause of a more severe course of COVID-19 and an increased risk of hospitalization in patients diagnosed with PD [50,51].

Based on a group of 10 patients with parkinsonism (two patients diagnosed with advanced disease including severe motor symptoms treated with levodopa), Antonini et al. concluded that elderly patients and patients with longer disease duration may be potentially more susceptible to COVID-19 and show a high mortality rate of 40%. The mortality rate was even higher (reaching 50%) among the four patients who required intensive treatment with deep brain stimulation or levodopa infusions [51].

Fasano et al. collected information from 21 centers in Italy, Iran, Spain, and the United Kingdom on a total of 117 patients with an average age of 71.4 years suffering from PD and receiving different treatment, who suffered from COVID-19 infection [50,52]. All the data were gathered through a phone survey and therefore subjected to subjective interpretation [52]. The report indicated an overall mortality rate of 20% and also confirmed the role of known risk factors for COVID-19 such as age and hypertension [50,52]. However, a significant limitation of this study was the qualification of patients not only on the basis of a positive PCR test, but also on the basis of compatible symptoms and exposure to SARS-CoV-2 [52].

Fasano et al. also gathered and analyzed data from 1486 PD patients and their 1207 family members as a control group [53]. As in the previous study, clinical information was obtained via phone survey. In this study, 105 patients with PD and 92 from the control group had been diagnosed with COVID-19. Six patients with PD and seven from the control group died due to the infection. Patients who had COVID-19 and PD were also more likely to suffer from obstructive pulmonary disease, obesity, and vitamin D deficiency [5,53,54,55]. Shortness of breath and the necessity for hospitalization were more frequently reported in COVID-19-positive patients as well [53]. Even though it has been hypothesized that anti-PD treatments such as levodopa, entacapone, and amantadine may have a protective effect against COVID-19, numerous studies failed to confirm their significance [50,53,56,57,58].

Vignatelli et al. reported a study in patients with a diagnosis of PD and parkinsonism, including criteria of age, sex, place of residence, and Charlson Index (used to predict 10-year survival in patients with multiple comorbidities) [54]. The study criteria were met by 696 patients with PD (mean age of 75 years) and 184 patients (average age of 80.5 years) with symptoms of parkinsonism, while 8590 people were appointed as a control group. During the three-month follow-up, it was determined that the rate of hospitalization of patients with COVID-19 and parkinsonism was about 3.3%, whereas in a group diagnosed with PD it oscillated around 0.6% and was not substantially different from the control group (0.7%). However, when statistical analysis was adjusted to include the comorbidities, patients with parkinsonism showed more than a three times higher risk (hazard ratio 3.3) compared to the control group. Interestingly, the hazard ratio in the PD group was equal to 0.8 [54,59]. The risk of death during or after hospitalization was approximately 35% in all the group of patients [54].

Sainz-Amo R. performed a retrospective case–control study on COVID-19 and PD patients. The report included 211 patients with PD. Thirty-nine of those were COVID-19-positive and 172 COVID-19-negative. The rate of comorbidities was similar in both groups except for dementia, which was more common in the COVID-19-positive group (36% vs 14%). Among COVID-19-positive patients, 10 (26%) experienced mild symptoms, 21 (54%) required hospitalization, and 8 (21%) cases were fatal. The study showed no significant differences between PD patients with or without COVID-19 in terms of age, disease duration, and treatment (including advanced PD therapies such as deep brain stimulation and levodopa intestinal gel), besides the observation that patients with PD and COVID-19 were less frequently treated with dopamine agonist [60]. Since there are discrepancies between the results of this study and data published earlier [51] regarding the relationship between advanced PD therapies and the severity of COVID-19, the authors suggest further prospective studies [60].

Xu et al. described manifestations of COVID-19 in a population of US patients with PD. The cohort included 46 (17 female and 29 male) patients with suspected or confirmed COVID-19. Clinical information was gathered through a telephone and online survey, as well as from data that patients reported to their movement disorder specialists. As in the general population, symptoms ranged from asymptomatic carriers (1 patient), mild (18 patients), and moderate (14). Seven patients required hospitalization, and six patients died. Fatal cases occurred in patients between the ages of 69 and 75, and five of the six were men. The most common reported symptoms were fever, chills, fatigue, cough, weight loss, and muscle pain. Worsening and occurrence of new motor and non-motor symptoms, such as dyskinesia, rigidity, balance disorder, anxiety, depression, and insomnia, were reported as well. Contrary to previous reports, the study revealed no significant difference in the number of deaths between COVID-19-positive patients treated with dopamine agonists, and those who were not. However, all six patients receiving amantadine in the course of COVID-19 survived [61]. Since amantadine has antiviral properties against influenza A strains, and there are few studies that support amantadine’s effects against COVID-19 [58,62,63], the authors recommend further investigation. The study concludes that there is no strong evidence that PD is an independent risk factor for a severe clinical course of COVID-19 and death [61].

Zhang et al. performed an analysis of the case fatality rate (CFR) in COVID-19 patients with PD. The study compared COVID-19 CFR in patients with PD with a large population matched via the TriNetX (Cambridge, MA, USA) COVID-19 research database and network. Logistic regression (adjusted for age, sex, and race) was applied in statistical analysis and revealed that PD patients had a significantly higher risk of dying from COVID-19 in comparison to the non-PD group (odds ratio 1.27). However, the database used lacks information on key comorbidities and potential recovery, which constitutes the main limitation of the aforementioned study [64].

Cosentino et al. reviewed medical dossiers of 181 patients (107 men and 74 women) with PD who received one or two doses of Pfizer/BioNtech (Pfizer, New York, NY, USA; BioNTech, Mainz, Germany) or Sinopharm (Shanghai, China) vaccine [37]. Out of 178 patients vaccinated with two doses (177 Pfizer/BioNTech and 1 Sinopharm) and 3 patients vaccinated with only one dose of Pfizer/BioNTech vaccine, only 2 subjects (1.1%) reported worsening of motor PD symptoms. Symptoms lasted a few days in the first case and about 2 weeks in the second case. In both patients the symptoms improved/resolved spontaneously without modification of anti-PD treatment.

The initial reports at the beginning of pandemic suggested that, due to its antiviral properties, amantadine (applied in PD treatment) might be effective against COVID-19 [58,62,63,65]. Amantadine interferes with viroporin protein channels responsible for the release of RNA viruses, such as SARS-CoV-2, from the infected cells [50]. Some authors even implied that patients with PD who are regularly treated with amantadine should be free of COVID-19 [65]. Since cases of PD patients infected with SARS-CoV-2 were reported, the protective role of amandine is questionable and requires further investigation [65,66].

Recent studies suggest that vitamin D may have a potential antiviral effect and may play a significant role in managing respiratory diseases. People with PD are likely to be vitamin D deficient; supplementation may help to improve the motor and non-motor parkinsonian symptoms. It seems that daily dose of 2000–5000 international units (IU) of the vitamin D3 in patients with PD may help to reduce the risk and severity of COVID-19, but this requires more research [5,55].

Regarding the severity of COVID-19 in patients with PD, the existing scientific evidence is contradictory, and the quality of published data is very diverse. The primary series reporting small numbers of patients pointed to a possible link between advanced PD and more a severe clinical course of SARS-CoV-2 infection. Similar results were obtained for a cohort of PD patients, in whom the severity of COVID-19 infection was compared to a database-matched control. However, the latter study failed to adjust the statistical analysis in terms of possible comorbidities in the case control group. The remaining cited manuscripts, based on relatively large study cohorts, did not reveal any significant differences between PD and non-PD patients in COVID-19 outcomes (length of hospital stay, fatal cases). Interestingly, the results of one study (Vignatelli et al.) specified that individuals with parkinsonism, but not PD, were at a higher risk of prolonged hospitalization compared to the control group; the mortality rates were comparable in all groups of patients [54]. Therefore, it seems reasonable to doubt whether PD alone may constitute an independent risk factor of severe COVID-19 infection; it is rather the constellation of PD/parkinsonism and multiple co-morbidities (also age-related) that plays a significant role. In addition, after initial delight on the promising role of both the amantadine and dopamine agonists in preventing complications and serious COVID-19-related health events, larger series failed to unequivocally prove their clinical effect.

Literature data relating possible exacerbations of PD/parkinsonian symptoms with COVID-19 infection are very scarce and mostly based on subjective impressions coming from phone/internet surveys, or data reported to caretakers by the patients. Some authors reported worsening and the occurrence of new motor and non-motor symptoms in individuals affected by SARS-CoV-2. However, not only the infection itself, but also the concept of dopaminergic adaptation to stressful stimulus, such as COVID-19, may be partially responsible for worse functional and mental states, at least in some PD patients.

## 4. Conclusions

In the past months, SARS-CoV-2 has become one of the major research interests, particularly due to its devastating impact on global healthcare and relatively high mortality rates among different populations. Like the already known representatives of the Coronaviridae family, SARS-CoV-2 has been proven to have neurotropic properties. The virus seems to exert harmful effects either in a direct manner, spreading through olfactory and gastrointestinal nervous pathways, or by evoking an inflammatory response. Some authors postulate the role of gut microbiota dysbiosis as a trigger for blood–brain barrier impairment and further accumulation of alpha-synuclein in the cerebral structures. Cytoplasmic build-up of the alpha-synuclein is postulated to promote neurodegeneration in the nigrostriatal system of patients affected by PD. Although parkinsonism is mainly associated with motor dysfunction, the hyposmia and hypogeusia that are common both for the prodromal PD and COVID-19 point to the fact that SARS-CoV-2 may have direct access to brain regions relevant for PD pathogenesis. The mutual impact of COVID-19 and PD on symptomatology of both diseases constitutes another interesting study topic. Despite the initial assumptions that, as a mainly elderly population suffering from rigidity of respiratory muscles, impairment of cough reflex and dyspnea, PD patients would experience more aggressive courses of COVID-19, the published scientific reports contain mutually exclusive data that require further investigation and meta-analysis.

## Data Availability

Not applicable.

## References

[1] Lukiw W.J. (2021). SARS-CoV-2, the Angiotensin Converting Enzyme 2 (ACE2) Receptor and Alzheimer’s Disease. J. Alzheimers Dis. Parkinsonism.

[2] Parry A.H., Wani A.H., Yaseen M. (2020). Neurological Dysfunction in Coronavirus Disease-19 (COVID-19). Acad. Radiol..

[3] Danilenko V., Devyatkin A., Marsova M., Shibilova M., Ilyasov R., Shmyrev V. (2021). Common Inflammatory Mechanisms in COVID-19 and Parkinson’s Diseases: The Role of Microbiome, Pharmabiotics and Postbiotics in Their Prevention. J. Inflamm. Res..

[4] Thakur K.T., Miller E.H., Glendinning M.D., Al-Dalahmah O., A Banu M., Boehme A.K., Boubour A.L., Bruce S.S., Chong A.M., Claassen J. (2021). COVID-19 neuropathology at Columbia University Irving Medical Center/New York Presbyterian Hospital. Brain.

[5] Carter S.J., Baranauskas M.N., Fly A.D. (2020). Considerations for Obesity, Vitamin D, and Physical Activity Amid the COVID-19 Pandemic. Obesity.

[6] Semerdzhiev S.A., Fakhree M.A.A., Segers-Nolten I., Blum C., Claessens M.M.A.E. (2021). Interactions between SARS-CoV-2 N-Protein and α-Synuclein Accelerate Amyloid Formation. ACS Chem. Neurosci..

[7] Satheesh N.J., Salloum-Asfar S., Abdulla S.A. (2021). The Potential Role of COVID-19 in the Pathogenesis of Multiple Sclerosis—A Preliminary Report. Viruses.

[8] Gordon M.N., Heneka M.T., Le Page L.M., Limberger C., Morgan D., Tenner A.J., Terrando N., Willette A.A., Willette S.A. (2021). Impact of COVID-19 on the Onset and Progression of Alzheimer’s Disease and Related Dementias: A Roadmap for Future Research. Alzheimer’s Dement..

[9] Bouali-Benazzouz R., Benazzouz A. (2021). Covid-19 Infection and Parkinsonism: Is There a Link?. Mov. Disord..

[10] Meng L., Shen L., Ji H.-F. (2019). Impact of infection on risk of Parkinson’s disease: A quantitative assessment of case-control and cohort studies. J. Neurovirology.

[11] Beauchamp L.C., Finkelstein D.I., Bush A.I., Evans A.H., Barnham K.J. (2020). Parkinsonism as a Third Wave of the COVID-19 Pandemic?. J. Park. Dis..

[12] Brundin P., Nath A., Beckham J.D. (2020). Is COVID-19 a Perfect Storm for Parkinson’s Disease?. Trends Neurosci..

[13] Poewe W., Seppi K., Tanner C.M., Halliday G.M., Brundin P., Volkmann J., Schrag A.E., Lang A.E. (2017). Parkinson disease. Nat. Rev. Dis. Primers.

[14] Ravenholt R., Foege W. (1982). 1918 Influenza, Encephalitis Lethargica, Parkinsonism. Lancet.

[15] Eldeeb M.A., Hussain F.S., Siddiqi Z.A. (2020). COVID-19 infection may increase the risk of parkinsonism–Remember the Spanish flu?. Cytokine Growth Factor Rev..

[16] Ayele B.A., Demissie H., Awraris M., Amogne W., Shalash A., Ali K., Zenebe Y., Tafesse A., Rao C.P.V. (2021). SARS-COV-2 induced Parkinsonism: The first case from the sub-Saharan Africa. Clin. Park. Relat. Disord..

[17] Ghosh R., Ray A., Roy D., Das S., Dubey S., Benito-León J. (2021). Parkinsonism with akinetic mutism following osmotic demyelination syndrome in a SARS-CoV-2 infected elderly diabetic woman: A case report. Neurología.

[18] Ong T.L., Nor K.M., Yusoff Y., Sapuan S. (2021). COVID-19 Associated Acute Necrotizing Encephalopathy Presenting as Parkinsonism and Myorhythmia. J. Mov. Disord..

[19] Alexoudi A., Gatzonis S. (2018). Parkinson’s disease pathogenesis, evolution and alternative pathways: A review. Rev. Neurol..

[20] Feigin V.L., Nichols E., Alam T., Bannick M.S., Beghi E., Blake N., Culpepper W.J., Dorsey E.R., Elbaz A., Ellenbogen R.G. (2019). Global, regional, and national burden of neurological disorders, 1990–2016: A systematic analysis for the Global Burden of Disease Study 2016. Lancet Neurol..

[21] Tarlinton R.E., Martynova E., Rizvanov A.A., Khaiboullina S., Verma S. (2020). Role of Viruses in the Pathogenesis of Multiple Sclerosis. Viruses.

[22] Braak H., de Vos R.A., Bohl J., Del Tredici K. (2006). Gastric α-synuclein immunoreactive inclusions in Meissner’s and Auerbach’s plexuses in cases staged for Parkinson’s disease-related brain pathology. Neurosci. Lett..

[23] Boika A.V. (2020). A Post-COVID -19 Parkinsonism in the Future?. Mov. Disord..

[24] Idrees D., Kumar V. (2021). SARS-CoV-2 spike protein interactions with amyloidogenic proteins: Potential clues to neurodegeneration. Biochem. Biophys. Res. Commun..

[25] Bantle C.M., Phillips A.T., Smeyne R.J., Rocha S.M., Olson K.E., Tjalkens R.B. (2019). Infection with mosquito-borne alphavirus induces selective loss of dopaminergic neurons, neuroinflammation and widespread protein aggregation. NPJ Park. Dis..

[26] Lautenschläger J., Kaminski C.F., Schierle G.S.K. (2017). alpha-Synuclein – Regulator of Exocytosis, Endocytosis, or Both?. Trends Cell Biol..

[27] Polymeropoulos M.H., Lavedan C., Leroy E., Ide S.E., Dehejia A., Dutra A., Pike B., Root H., Rubenstein J., Boyer R. (1997). Mutation in the α-Synuclein Gene Identified in Families with Parkinson’s Disease. Science.

[28] Ibáñez P., Bonnet A.-M., Débarges B., Lohmann E., Tison F., Agid Y., Dürr A., Brice A., Pollak P. (2004). Causal relation between alpha-synuclein gene duplication and familial Parkinson’s disease. Lancet.

[29] Pavel A., Murray D.K., Stoessl A.J. (2020). COVID-19 and selective vulnerability to Parkinson’s disease. Lancet Neurol..

[30] Beatman E.L., Massey A., Shives K.D., Burrack K.S., Chamanian M., Morrison T.E., Beckham J.D. (2016). Alpha-Synuclein Expression Restricts RNA Viral Infections in the Brain. J. Virol..

[31] Marreiros R., Müller-Schiffmann A., Trossbach S.V., Prikulis I., Hänsch S., Weidtkamp-Peters S., Moreira A.R., Sahu S., Soloviev I., Selvarajah S. (2020). Disruption of cellular proteostasis by H1N1 influenza A virus causes α-synuclein aggregation. Proc. Natl. Acad. Sci. USA.

[32] Chaudhry Z.L., Klenja D., Janjua N., Cami-Kobeci G., Ahmed B.Y. (2020). COVID-19 and Parkinson’s Disease: Shared Inflammatory Pathways Under Oxidative Stress. Brain Sci..

[33] Hoffmann M., Kleine-Weber H., Schroeder S., Krüger N., Herrler T., Erichsen S., Schiergens T.S., Herrler G., Wu N.-H., Nitsche A. (2020). SARS-CoV-2 Cell Entry Depends on ACE2 and TMPRSS2 and Is Blocked by a Clinically Proven Protease Inhibitor. Cell.

[34] Benigni A., Cassis P., Remuzzi G. (2010). Angiotensin II revisited: New roles in inflammation, immunology and aging. EMBO Mol. Med..

[35] Hirano T., Murakami M. (2020). COVID-19: A New Virus, but a Familiar Receptor and Cytokine Release Syndrome. Immunity.

[36] Wiese O., Allwood B., Zemlin A. (2020). COVID-19 and the renin-angiotensin system (RAS): A spark that sets the forest alight?. Med. Hypotheses.

[37] Bhaskar S., Sinha A., Banach M., Mittoo S., Weissert R., Kass J.S., Rajagopal S., Pai A.R., Kutty S. (2020). Cytokine Storm in COVID-19—Immunopathological Mechanisms, Clinical Considerations, and Therapeutic Approaches: The REPROGRAM Consortium Position Paper. Front. Immunol..

[38] Wright J.W., Kawas L.H., Harding J.W. (2013). A Role for the Brain RAS in Alzheimer’s and Parkinson’s Diseases. Front. Endocrinol..

[39] Reardon K.A., Mendelsohn F.A.O., Chai S.Y., Horne M.K. (2000). The angiotensin converting enzyme (ACE) inhibitor, perindopril, modifies the clinical features of Parkinson’s disease. Aust. New Zealand J. Med..

[40] Jenkins T.A., Wong J.Y.F., Howells D.W., Mendelsohn F.A.O., Chai S.Y. (2002). Effect of chronic angiotensin-converting enzyme inhibition on striatal dopamine content in the MPTP-treated mouse. J. Neurochem..

[41] Baizabal-Carvallo J.F., Alonso-Juarez M. (2020). The Link between Gut Dysbiosis and Neuroinflammation in Parkinson’s Disease. Neuroscience.

[42] Zuo T., Zhang F., Lui G.C.Y., Yeoh Y.K., Li A.Y.L., Zhan H., Wan Y., Chung A.C.K., Cheung C.P., Chen N. (2020). Alterations in Gut Microbiota of Patients With COVID-19 During Time of Hospitalization. Gastroenterology.

[43] Zuo T., Liu Q., Zhang F., Lui G., Tso E., Yeoh Y.K., Chen Z., Boon S., Chan F.K.L., Chan P. (2020). Depicting SARS-CoV-2 faecal viral activity in association with gut microbiota composition in patients with COVID-19. Gut.

[44] Yamamoto S., Saito M., Tamura A., Prawisuda D., Mizutani T., Yotsuyanagi H. (2021). The human microbiome and COVID-19: A systematic review. PLoS ONE.

[45] Cilia R., Bonvegna S., Straccia G., Andreasi N.G., Elia A.E., Romito L.M., Devigili G., Cereda E., Eleopra R. (2020). Effects of COVID -19 on Parkinson’s Disease Clinical Features: A Community-Based Case-Control Study. Mov. Disord..

[46] Erro R., Buonomo A.R., Barone P., Pellecchia M.T. (2021). Severe Dyskinesia After Administration of SARS-CoV2 mRNA Vaccine in Parkinson’s Disease. Mov. Disord..

[47] Balestrino R., Schapira A.H. (2020). Parkinson disease. Eur. J. Neurol..

[48] Helmich R.C., Bloem B.R. (2020). The Impact of the COVID-19 Pandemic on Parkinson’s Disease: Hidden Sorrows and Emerging Opportunities. J. Park. Dis..

[49] Brown E.G., Chahine L.M., Goldman S.M., Korell M., Mann E., Kinel D.R., Arnedo V., Marek K.L., Tanner C.M. (2020). The Effect of the COVID-19 Pandemic on People with Parkinson’s Disease. J. Park. Dis..

[50] Rejdak K., Grieb P. (2020). Adamantanes might be protective from COVID-19 in patients with neurological diseases: Multiple sclerosis, parkinsonism and cognitive impairment. Mult. Scler. Relat. Disord..

[51] Antonini A., Leta V., Teo J., Chaudhuri K.R. (2020). Outcome of Parkinson’s Disease Patients Affected by COVID-19. Mov Disord..

[52] Fasano A., Elia A.E., Dallocchio C., Canesi M., Alimonti D., Sorbera C., Alonso-Canovas A., Pezzoli G. (2020). Predictors of COVID-19 outcome in Parkinson’s disease. Park. Relat. Disord..

[53] Fasano A., Cereda E., Barichella M., Cassani E., Ferri V., Zecchinelli A.L., Pezzoli G. (2020). COVID -19 in Parkinson’s Disease Patients Living in Lombardy, Italy. Mov. Disord..

[54] Vignatelli L., Zenesini C., Belotti L.M.B., Baldin E., Bonavina G., Calandra-Buonaura G., Cortelli P., Descovich C., Fabbri G., Giannini G. (2021). Risk of Hospitalization and Death for COVID -19 in People with Parkinson’s Disease or Parkinsonism. Mov. Disord..

[55] Jakovac H. (2020). COVID-19 and vitamin D—Is there a link and an opportunity for intervention?. Am. J. Physiol. Metab..

[56] Nataf S. (2020). An alteration of the dopamine synthetic pathway is possibly involved in the pathophysiology of COVID-19. J. Med Virol..

[57] Gordon D.E., Jang G.M., Bouhaddou M., Xu J., Obernier K., O’Meara M.J., Guo J.Z., Swaney D.L., Tummino T.A., Huettenhain R. (2020). A SARS-CoV-2-Human Protein-Protein Interaction Map Reveals Drug Targets and Potential Drug-Repurposing. bioRxiv.

[58] Smieszek S.P., Przychodzen B.P., Polymeropoulos M.H. (2020). Amantadine disrupts lysosomal gene expression: A hypothesis for COVID19 treatment. Int. J. Antimicrob. Agents.

[59] Ko J.Y., Danielson M.L., Town M., Derado G., Greenlund K.J., Kirley P.D., Alden N.B., Yousey-Hindes K., Anderson E.J., A Ryan P. (2021). Risk Factors for Coronavirus Disease 2019 (COVID-19)–Associated Hospitalization: COVID-19–Associated Hospitalization Surveillance Network and Behavioral Risk Factor Surveillance System. Clin. Infect. Dis..

[60] Sainz-Amo R., Baena-Álvarez B., Pareés I., Sánchez-Díez G., Pérez-Torre P., López-Sendón J.L., Fanjul-Arbos S., Monreal E., Corral-Corral I., García-Barragán N. (2021). COVID-19 in Parkinson’s disease: What holds the key?. J. Neurol..

[61] Xu Y., Surface M., Chan A.K., Halpern J., Vanegas-Arroyave N., Ford B., Feeney M.P., Kwei K.T., Katus L.E., Kuo S.-H. (2021). COVID-19 manifestations in people with Parkinson’s disease: A USA cohort. J. Neurol..

[62] Abreu G.E.A., Aguilar M.E.H., Covarrubias D.H., Duran F.R. (2020). Amantadine as a drug to mitigate the effects of COVID-19. Med. Hypotheses.

[63] Aranda-Abreu G.E., Aranda-Martínez J.D., Araújo R. (2021). Use of Amantadine in a Patient with SARS-CoV-2. J. Med. Virol..

[64] Zhang Q., Schultz J.L., Aldridge G.M., Simmering J.E., Narayanan N.S. (2020). Coronavirus Disease 201 9 Case Fatality and Parkinson’s Disease. Mov. Disord..

[65] Wiwanitkit V. (2020). Amantadine, COVID-19 and Parkinsonism. Arch. Med. Res..

[66] Borra A.C. (2020). Does amantadine have a protective effect against COVID-19?. Neurol. Neurochir. Polska.

